# Crosstalk between Innate and Adaptive Cells on Allergic Process

**DOI:** 10.1155/2012/720568

**Published:** 2012-12-26

**Authors:** Maria Leite-de-Moraes, Hamida Hammad, Michel Dy

**Affiliations:** ^1^CNRS (Centre National de la Recherche Scientifique), UMR (Unité Mixte de Recherche) 8147, Université Paris Descartes, Sorbonne, 75743 Paris, France; ^2^Laboratory of Immunoregulation and Mucosal Immunology, Ghent University, 9000 Ghent, Belgium

As guest editors of this special issue of the *Journal of Allergy*, we are pleased to introduce both original article, and reviews that address pertinent questions associated with a critical view of the current knowledge of *crosstalk between innate and adaptive cells on allergic process*.

Innate cells play a major role in the first line of defense against invading organisms and environmental challenges. However, in some cases they are deflected from their primary role to participate, in association with adaptive cells, on allergic process. Innate cells are constantly in contact with anodyne environmental molecules that for still not well understood reasons trigger allergic responses in a number of individuals. Both prevalence and severity of allergies are increasing worldwide. Allergic asthma is a disease characterized by a chronic airway inflammation with the infiltration of Th2 cells and eosinophils, airway obstruction, and remodeling. Notably, 250 000 people die of asthma every year. About 10% to 20% of the population are allergic to house dust mite (HDM), the causative agent of some types of atopic dermatitis or allergic asthma. Food allergy also represents a public health problem associated with diminished quality of life. The symptoms range from mild erythema and pruritus to anaphylaxis. Milk, eggs, fish, or peanuts allergies are very common in the general population, and the latter allergen is highly associated with anaphylaxis.

Innate cells are the first-line barrier in contact with allergens in skin, airways, and gut. Therefore, they will send messages by cell-to-cell contact or cytokines to positively or negatively influence adaptive cells. Epithelial cells can express many pattern recognition receptors (PPRs) to detect and respond to pathogen-associated molecular patters (PAMPs) currently found in microbes like mites or damaged tissues. This stimulation will induce the production of cytokines as TGF-*β* (transforming growth factor beta), TSLP (thymic stomal lymphopoietin), Mip2 (macrophage inflammatory protein 2), IL-1 (interleukin-1), IL-25, IL-33, or GM-CSF (granulocyte-macrophage colony-stimulating factor) that will act on other cells not only of the innate but also of the adaptive immunity [1]. In brief, TSLP, GM-CSF, IL-25, and IL-33 will activate dendritic cells to prime Th2 response by inhibiting the production of the Th1-polarizing cytokine IL-12, by inducing chemokines that attract Th2 cells or by favoring the development of Th2 cells through the upregulation of OX40L. 

Dendritic cells (DCs) are the most important antigen presenting cells (APC) that will induce T-cell differentiation. However, this major capacity can vary depending on their distinct subsets. Several markers are used to distinguish these subsets, but the most simple discrimination is based on the level of expression of CD11c and CD11b. Conventional (c) DCs express high levels of CD11c compared with CD11c^dim^ plasmacytoid (p) DCs [2]. Despite the fact that great advance on the understanding of the biology of DC subsets was already obtained, further studies are necessary to clarify how allergic sensitization is induced and the implication of the distinct DC subsets.

In addition to DC, innate cells as basophils can also influence adaptive immune responses. Basophils amplify allergic Th2 cells initiated by dendritic cells via a nonredundant role because of their capacity to generate IL-4 rapidly and efficiently on exposure to an increasing number of stimuli including IgE [3]. For instance, it has been established that basophils can be targeted in an IgE-independent manner by allergen proteases, such as papain, which promotes their recruitment and stimulation in draining lymph nodes, initiating a Th2 immune response. Moreover, basophils directly respond to IL-33 by producing substantial amounts of the Th2 promoting factors IL-4, IL-6, and histamine [4]. It is notably that histamine will promote IL-4 production by invariant natural killer T (iNKT) cells [5].

iNKT cells are in the very frontier between innate and adaptive immune responses. They constitute a distinctive population of mature T lymphocytes that produce a broad range of cytokines few minutes after stimulation allowing them to modulate both innate and acquired immunity in a large spectrum of inflammatory diseases. They express a highly restricted T-cell receptor (TCR) repertoire composed of a single-invariant V*α*14J*α*18 chain in mice and a V*α*24J*α*18 chain in humans, preferentially paired with limited TCR V*β* chains. In contrast to conventional T cells that recognize peptides, iNKT cells recognize glycolipids presented by CD1d expressing Ag-presenting cells (APCs), such as dendritic cells (DCs). The major iNKT cell subset promptly and massively produces IL-4 and IFN-*γ*. iNKT cells enhanced the severity of asthmatic symptoms in experimental models, namely, airway eosinophilia, hyperreactivity, Th2 cytokine production, and mucus and IgE secretion and are implicated on food allergy [6–8]. Moreover, iNKT cells may have similar effects in humans since they are enhanced in bronchoalveolar lavage fluid of asthmatic patients [9, 10]. In addition to acting as effector cells, iNKT lymphocytes can also be considered as a novel biomarker for some pathologies. Indeed, it was recently provided the first demonstration in humans that early postallogeneic HSCT (hematopoietic stem cell transplantation) donor-derived iNKT cell recovery can be used as a new predictive marker of acute GVHD (graft-versus-host disease) with preserved GVL (graft versus leukemia) effect and improved overall survival [11].

Recently a new iNKT cell subset that preferentially produces IL-17 were discovered [12, 13]. This iNKT17 subset derives from a alternative thymic pathway of differentiation dependent on the transcription factor ROR*γ*t that, in contrast to the mainstream IL-4-producing ROR*γ*t^neg^ iNKT cell subset, maintains the expression of this transcription factor in the periphery [13]. iNKT17 cells are present in the lung and can exacerbate allergic airway inflammation. IL-17-producing iNKT cells were also observed in humans [14]. Further studies are in progress to better characterize the crosstalk of these cells with epithelial cells, DC, basophils and conventional T and B cells in allergic responses.

In this special issue, S. Schnyder-Candrian et al. reported that neutrophil inhibitory factor (NIF) can preferentially block the transmigration of eosinophils across endothelial cell monolayers. The inhibitory effect was confirmed *in vivo* in an experimental asthma model. These findings clearly suggest that NIF has an antiallergic effect and show a new facet of this molecule primarily implicated on neutrophil migration. In addition to endothelial cells, keratinocytes represent not only a mechanical barrier but also an important source of cytokines and chemokines as reviewed by the French group from Poitiers. In fact, F. Bernard et al. emphasized the critical role of cytokines, namely, IL-4, IL-13, IL-17, IL-22, and TNF*α*, in keratinocytes and the implication of this crosstalk in atopic dermatitis (AD) and psoriasis. They mounted an elegant model of reconstructed human epidermis (RHE) that mimics human skin. This group demonstrated that specific sets of cytokines-induced AD-like or psoriasis-like phenotype in human keratinocytes. The theme is, therefore, expertly reviewed and discussed in this paper based on their own experience and those from others groups.

As commented before, DCs are professional APC critically implicated on the sensitization phase of allergic responses acting as a bridge between innate and adaptive immunity. S. Awasthi et al. provide an overview of the lung DC developmental programming. It is noteworthy that lung DCs develop after birth. Considering the frequent exposure to allergens, pathogens and environmental chemicals during the critical window that encompass lung DC maturation, authors stress the fact that these factors potentially influence DC subsets with long-term respiratory and immunological consequences. The study of lung DC development in neonates is not possible in humans, but authors have an important experience with baboon models that could help to better identify the basis of childhood asthma, and here they discuss the more recent findings concerning this central issue of DC differentiation and allergen sensitization. 

 Another actor in this crosstalk between innate and adaptive responses in allergies are B cells and antibodies. The term atopy was first used by Coca and Cooke in 1923 [15]. At this time, atopy was associated with hypersensitiveness but not necessarily with immunoglobulin (Ig) production. In this issue, W. Williams et al. discuss the contribution of allergen-specific IgG to the development of Th2-mediated airway inflammation. This paper discusses the place of immunoglobulin in the asthmatic process highlighting the importance of IgG and Fc*γ*Rs signaling. The authors propose a model whereby allergen-specific IgG promotes the expansion of secondary Th2 responses through ligation of FcyRs on innate cells. They also address the question concerning the implication of B cells and FcyRs on innate cells and their contribution to allergic immune responses.

 We could not conclude this issue without discussing the implication of regulatory T cells on allergic process. The Portuguese group of Lisbon leaded by L. Graça addressed this point. Allergic process represents ultimately exacerbated responses to a given antigen that is not tolerated by the organism. The authors discuss why some antigens are tolerized by the immune system and why others are “seen” as allergens. Many questions discussed in this paper are still without answers but they stimulate the continuing efforts to understand the crosstalk between innate and adaptive cells on allergic process and potentially provide better care to patients.

 Finally, this special issue provides an instructive overview of the different actors from both innate and adaptive immune cells and their crosstalk on allergic process. We would like to thank the authors' contributions, and we hope that this issue will bring new ideas to improve the scientific research against this health public scourge named allergy.

*Maria Leite-de-Moraes* *Maria Leite-de-Moraes* *Hamida Hammad* *Hamida Hammad* *Michel Dy* *Michel Dy*
